# In vivo evaluation of selenium-tellurium based nanoparticles as a novel treatment for bovine mastitis

**DOI:** 10.1186/s40104-024-01128-y

**Published:** 2024-12-20

**Authors:** Ludmila Kosaristanova, Zuzana Bytesnikova, Tatiana Fialova, Jana Pekarkova, Pavel Svec, Frantisek Ondreas, Vendula Jemelikova, Andrea Ridoskova, Peter Makovicky, Ladislav Sivak, Monika Dolejska, Monika Zouharova, Petr Slama, Vojtech Adam, Kristyna Smerkova

**Affiliations:** 1 Department of Chemistry and Biochemistry, Faculty of AgriSciences, Mendel University in Brno, Zemědělská 1665/1, Brno, 613 00 Czech Republic; 2 Central European Institute of Technology, Brno University of Technology, Purkyňova 656/123, Brno, 612 00 Czech Republic; 3 Department of Microelectronics, Faculty of Electrical Engineering and Communication, Brno University of Technology, Technická 3058/10, Brno, 616 00 Czech Republic; 4 Contipro a.s., Dolní Dobrouč 401, Dolní Dobrouč, 561 02 Czech Republic; 5 Department of Histology and Embryology, Faculty of Medicine, University of Ostrava, Syllabova 9, Ostrava - Vítkovice, 700 03 Czech Republic; 6 Cancer Research Institute, Biomedical Research Center of the Slovak Academy of Sciences, Dúbravská cesta 9, Bratislava, 845 05 Slovak Republic; 7 Central European Institute of Technology, University of Veterinary Sciences Brno, Palackého tř. 1946/1, Brno, 612 42 Czech Republic; 8 Department of Biology and Wildlife Diseases, Faculty of Veterinary Hygiene and Ecology, University of Veterinary Sciences Brno, Palackého tř. 1946/1, Brno, 612 42 Czech Republic; 9 Department of Microbiology, Faculty of Medicine, Charles University, Alej Svobody 76, Pilsen, 323 00 Czech Republic; 10 Division of Clinical Microbiology and Immunology, Department of Laboratory Medicine, The University Hospital Brno, Jihlavská 20, Brno, 625 00 Czech Republic; 11 Department of Infectious Diseases and Preventive Medicine, Veterinary Research Institute, Hudcova 296/70, Brno, 621 00 Czech Republic; 12 Laboratory of Animal Immunology and Biotechnology, Department of Animal Morphology, Physiology and Genetics, Faculty of AgriSciences, Mendel University in Brno, Zemědělská 1665/1, Brno, 613 00 Czech Republic

**Keywords:** Antibacterial, Biocompatibility, Heifer, Intramammary, Mammary gland, MRSA, Nanomaterial, Resistance, SeTe

## Abstract

**Background:**

Bovine mastitis is one of the main causes of reduced production in dairy cows. The infection of the mammary gland is mainly caused by the bacterium *Staphylococcus aureus,* whose resistant strains make the treatment of mastitis with conventional antibiotics very difficult and result in high losses. Therefore, it is important to develop novel therapeutic agents to overcome the resistance of mastitis-causing strains. In this study, novel selenium-tellurium based nanoparticles (SeTeNPs) were synthesized and characterized. Their antibacterial activity and biocompatibility were evaluated both in vitro and in vivo using a bovine model. A total of 10 heifers were divided into experimental and control groups (5 animals each). After intramammary infection with methicillin resistant *S. aureus* (MRSA) and the development of clinical signs of mastitis, a dose of SeTeNPs was administered to all quarters in the experimental group.

**Results:**

Based on in vitro tests, the concentration of 149.70 mg/L and 263.95 mg/L of Se and Te, respectively, was used for application into the mammary gland. Three days after SeTeNPs administration, MRSA counts in the experimental group showed a significant reduction (*P* < 0.01) compared to the control group. The inhibitory effect observed within the in vitro experiments was thus confirmed, resulting in the suppression of infection in animals. Moreover, the superior biocompatibility of SeTeNPs in the organism was demonstrated, as the nanoparticles did not significantly alter the inflammatory response or histopathology at the site of application, i.e., mammary gland, compared to the control group (*P* > 0.05). Additionally, the metabolic profile of the blood plasma as well as the histology of the main organs remained unaffected, indicating that the nanoparticles had no adverse effects on the organism.

**Conclusions:**

Our findings suggest that SeTeNPs can be used as a promising treatment for bovine mastitis in the presence of resistant bacteria. However, the current study is limited by its small sample size, making it primarily a proof of the concept for the efficacy of intramammary-applied SeTeNPs. Therefore, further research with a larger sample size is needed to validate these results.

**Supplementary Information:**

The online version contains supplementary material available at 10.1186/s40104-024-01128-y.

## Background

Bovine mastitis is considered to be one of the most prevalent diseases affecting milk production of high-yielding dairy cattle, leading to major economic losses due to poor milk quality, medical costs, and welfare of the animals themselves [1–3]. Mastitis is defined as inflammation of the mammary gland, where the most common signs of acute mastitis are the presence of red and swollen udder quarters, a change in the appearance and quality of the milk, deviation from the basal temperature in animals and decreased appetite. Also, behavioral changes in cows have been noted [4, 5].

In addition to the mechanical and chemical causes of the development of mastitis, microbial factors such as bacteria play a significant role in the aetiology of mastitis [6]. The contagious bacteria taking part in this process are pathogenic forms of Gram-positive *Staphylococcus aureus* and *Streptococcus agalactiae*, among others. These pathogens are especially important, because they are responsible for subclinical forms of mastitis, which are often very hard to detect and are commonly transmitted between the cattle through infected milk. In addition to the microorganisms mentioned above, primary environmental pathogens include coliform species such as Gram-negative *Escherichia coli*, *Klebsiella* spp*.*, *Enterobacter* spp*.* and *Pseudomonas* spp. [7, 8]. Nevertheless, based on epidemiological studies and mastitis surveillance efforts, *S. aureus* has been classified as the most prevalent causative agent of mastitis in numerous parts of the world [9]. Hence, antibiotics remain a crucial part of the treatment and management of mastitis. Since microorganisms have many adaptive mechanisms of action, the (over)use of antibiotics can lead to the emergence of antimicrobial resistance, thereby making the treatment of mastitis even more challenging [10]. There have also been reports of chronic biofilm infections, which are very difficult to eradicate with antibiotics. Biofilm formation with its very coherent constitution may therefore be a possible explanation for severe cases of mastitis that cannot be resolved by standard treatment [11].

In recent years, numerous publications have elucidated the role of resistant pathogens as causative agents of bovine mastitis [12]. Additionally, methicillin resistance has been reported in *S. aureus* isolates from cases of bovine mastitis [13, 14]. In light of concerns about antibiotic treatment failures, researchers have increasingly focused on developing novel therapeutic strategies in recent decades. Among these strategies, nanoparticles (NPs), both organic and inorganic, have emerged as a promising option. Several studies have demonstrated that various NPs can be effective in treating bovine mastitis infections [15–17]. For instance, silver (Ag) and copper (Cu) based NPs have displayed exceptional antimicrobial activity with low toxicity to mammary tissue in the treatment of mastitis [18–20]. However, there are ongoing concerns about the toxicity profile of these nanoparticles. Consequently, other NPs, such as selenium (Se) and tellurium (Te) based, are also being investigated as potential alternatives [21, 22]. NPs based on Se or Te are known for their outstanding antimicrobial properties and lower toxicity to humans compared to other metal NPs [23, 24]. The precise antibacterial mechanisms of various nanomaterials are not yet fully elucidated, but in the case of SeNPs, the three widely recognized mechanisms have been identified: the reactive oxygen species (ROS) production, interaction with and disruption of the cell membrane, and damage of biomacromolecules such as DNA and proteins [25–27]. ROS generation appears to be the primary mode of action, leading to oxidative damage of key biological macromolecules. This oxidative stress disrupts the bacterial cell membrane, causing leakage of cytoplasmic contents and ultimately resulting in bacterial cell death [28, 29]. Similarly, TeNPs exhibit antibacterial activity primarily through membrane disruption and ROS production [22, 30]. A key advantage of NPs is their ability to act via multiple mechanisms simultaneously, making it more difficult or impossible for bacteria to develop resistance. Since Se and Te belong to Group VIA of the periodic table, classified as chalcogens, they are presumed to exert biological effects such as anticancer properties and antioxidant activity in addition to antimicrobial effects [31, 32]. Moreover, Se is an essential component of selenoproteins, which play crucial roles in the body’s antioxidant defense mechanisms, primarily through enzymes like glutathione peroxidase and thioredoxin reductase. By harnessing their antioxidant properties, selenoproteins also play a key role in modulating inflammation [33, 34]. Therefore, they appear to be a promising and safe alternative in the treatment of various diseases, including bovine mastitis. Building upon previous research, our objective was to synthesize SeTeNPs and evaluate their in vitro efficacy against a mastitis strain of methicillin-resistant *S. aureus* (MRSA). Following this, we conducted an in vivo assessment in heifers, monitoring both the overall health status of the cattle and the therapeutic impact of the NPs on the infection.

## Materials and methods

### Synthesis and characterization of SeTeNPs

#### Synthesis of SeTeNPs

Selenous acid (1 mol/L; 0.4 mL) was combined with tellurous acid (1 mol/L; 0.4 mL), polyvinylpyrrolidone (PVP, 10%; 29 kDa; 10 mL; Merck, Germany), and ultrapure water (38.8 mL) in an Erlenmeyer flask. The mixture was stirred vigorously for 15 min and heated to 80 °C in an oil bath. Afterward, sodium borohydride (60 mg/mL; 1 mL; Merck, Germany) was quickly added, and the reaction was maintained at 80 °C for 1 h. The final product was washed 3 times with ultrapure water by centrifugation (15,000 × *g*, 30 min).

#### Characterization of SeTeNPs using transmission electron microscopy (TEM)

The sample was visualized by high-resolution transmission electron microscopy (HRTEM) Talos F200X (FEI, Hillsboro, OR, USA) operated at 200 kV with a maximum beam current of 1.0 nA. The lower amount of beam current was selected to avoid harming the sample. A Super-X EDS system with four silicon drift detectors was available to element mapping. The sample was deposited onto a gold grid coated with a carbon film. The ProcessDiffraction was employed for the measured selected area electron diffraction assessment [35].

#### Characterization of SeTeNPs using X-ray photoelectron spectroscopy (XPS)

XPS with monochromatic Al Kα X-ray radiation, emission current of 15 mA, and hybrid lens mode (Axis Supra, Kratos Analytical Manchester, UK), was used for the analysis of the SeTeNPs surface. High resolution spectra were determined with pass energy of 20 eV. The spectra were fitted using a combination of Gaussian–Lorentzian line shape in CasaXPS software version 2.3.22. All spectra were calibrated using C 1s peaks with a fixed value of 284.8 eV [35]. The Shirley algorithm was employed to establish the background of the spectra [36].

#### Characterization of SeTeNPs using X-ray diffraction (XRD)

For the analysis of powder XRD to determine crystalline phases, the SmartLab 3 kW diffractometer (Rigaku, Japan) was utilized. The measurements were performed using Bragg–Brentano geometry and Cu Kα radiation (λ = 0.154 nm) [37]. The diffractometer operated at a current of 30 mA and voltage of 40 kV. The scanning range for the diffraction patterns was set from 10° to 100°, with a step size of 0.02° and a scanning speed of 4°/min. The acquired data was then fitted using Rigaku PDXL2 software.

#### Determination of the Se and Te concentrations by atomic absorption spectrometry (AAS)

Analysis of the Se and Te content in NPs solutions was determined using a 240 FS AA atomic absorption spectrometer (Agilent Technologies, Santa Clara, CA, USA) with flame atomization (acethylene-air flame, oxygen flow 13.5 L/min and acethylene 2.0 L/min). Standard solution of Se and Te (1,000 mg/L; Merck, Germany) was used to preparation of the calibration solutions, which were acidified with 1% (w:w) concentrated HNO_3_. All solutions were prepared using demineralized water obtained with a Millipore Milli-Q system (Millipore, Bedford, MA, USA). NPs solutions were diluted by 5% HNO_3_. The wavelength for Se was 196 nm and for Te was 214.3 nm.

### In vitro testing of SeTeNPs biological activity

#### Cytotoxic properties of SeTeNPs on eukaryotic cell line

Spontaneously transformed aneuploidy immortal keratinocyte cell line from adult human skin (HaCaT) was cultured in DMEM medium with 10% fetal bovine serum, supplemented with penicillin (100 U/mL) and streptomycin (0.1 mg/mL). Cells were harvested, washed 4 times with phosphate-buffered saline (PBS, pH 7.4) and counted using Countess IIFL Automated Cell Counter (Life Technologies, Carlsbad, CA, USA). Cell viability was estimated using the MTT [3-(4,5-dimethylthiazol-2-yl)-2,5-diphenyltetrazolium bromide] assay. The suspension of 5,000 cells in 50 µL medium was added to each well of microtiter plates (E-plates 96), followed by incubation for 24 h at 37 °C with 5% CO_2_ to ensure the cell growth. The treatment was initiated after the cells reached ~60%–80% confluence and 50 µL of medium containing SeTeNPs in concentrations of 2.34/4.12–149.70/263.95 µg/L of Se/Te was employed. Treated cells were incubated for 24 h. Further, 10 µL of MTT (5 mg/mL in PBS) was added to the cells and the mixture was incubated at 37 °C for 4 h. MTT-containing medium was replaced by 100 µL of 99.9% dimethyl sulfoxide to dissolve MTT-formazan crystals and, after 5 min incubation, absorbance of the samples was measured at 570 nm (VersaMax microplate reader, Molecular Devices, Sunnyvale, CA, USA) [38].

#### Evaluation of inhibitory effect of SeTeNPs on bacteria

MRSA strain (isolated from mastitis in May 2017) was obtained from the Veterinary Research Institute, Czech Republic (a detailed description of the strain’s sensitivity to antimicrobials is included in the Supplementary information, Table S1). The bacterial strain was cultured on 5% Columbia blood agar (LMS, Czech Republic) at 37 °C overnight.

Minimum inhibitory concentration (MIC) of SeTeNPs against MRSA was determined by the broth microdilution method. MRSA strain was diluted in 2 × concentrated Mueller Hinton broth (Sigma Aldrich, USA) to achieve turbidity corresponding 0.5 McFarland units and then diluted 100 × to reach cell density 1–2 × 10^6^ CFU/mL. One hundred μL of prepared bacterial culture was placed in 96-well microplates and 100 μL of SeTeNPs at concentrations range 2.34/4.12–149.70/263.95 µg/L (diluted in sterile MilliQ water) was added. As a control, bacterial culture mixed with water was used. The absorbance reads at optical density with 620 nm were monitored at times 0 and 24 h by Multiscan (Thermo Scientific, USA), whereas the culture was incubated at 37 °C for 24 h [39]. MIC was identified as the lowest concentrations at which there was no visible growth of bacteria.

#### Cell morphology of MRSA after SeTeNPs treatment

MRSA culture was diluted in 2 × Mueller Hinton broth to reach turbidity 0.5 McFarland units and mixed with SeTeNPs to achieve subinhibitory concentration of 74.85/131.98 µg/L. As a control, bacterial culture mixed with water was used. These samples were cultured at 37 °C overnight. After incubation, the samples were centrifuged (1,000 × *g*, 5 min). One mL of PBS was added to the pellet and incubated at 37 °C/45 min/600 ×* g*. Then the samples were centrifuged (3,000 × *g*, 2 min) and washed 3 times with PBS. Glutaraldehyde (1%) was added to the pellet and incubated 30 min in the dark at room temperature of 22 °C. After incubation, the samples were washed 3 times by MilliQ water, when 1 mL of MilliQ water was added, incubated 10 min and centrifuged (3,000 × *g*, 2 min). The washed samples were dehydrated using an ascending ethanol series in range 40%–100% in several steps. Each time, the appropriate percentage of ethanol was added to the samples, incubated 5 min, and centrifuged (3,000 × *g*, 2 min). Samples with 100% ethanol were incubated for 5 and 15 min, washed 2 × with 100% ethanol and centrifuged (3,000 × *g*, 2 min). The morphology was examined by scanning electron microscopy (SEM) on a Tescan MAIA 3 equipped with a field emission gun (Tescan Ltd., Brno, Czech Republic). Best images were obtained using the In-Beam SE detector at working distance was approximately 3.00 mm and at 2 kV acceleration voltages. The 768 × 858 pixels images were obtained at 22,100-fold magnification covering sample area of 9.392 µm^2^. Full frame capture was performed in UH resolution mode and accumulation of image with image shift correction enabled, and it took about 0.5 min with the ∼1 µs/pixel dwell time. Spot size was set at 4.14 nm [40].

#### Fluorescence microscopy of MRSA after SeTeNPs treatment

The bacterial culture of MRSA with the optical density corresponding to 0.5 McFarland units in TSB (Tryptone soy broth, Oxoid, UK) was mixed with SeTeNPs to reach subinhibitory concentration 74.85/131.98 µg/L. The samples were incubated under rotation at 37 °C overnight. As a control, bacterial culture mixed with water was used. After incubation, bacterial cells were purified by centrifugation and the TSB was replaced with PBS. To stain the bacteria, LIVE/DEAD BacLight Bacterial Viability and Counting Kit (ThermoFisher, USA) was used according to manufacturer’s instructions. The kit contains fluorescence dyes SYTO-9, which stains all cells, and propidium iodide (PI), which stains only the dead cells. After incubation, the samples were observed by OLYMPUS IX71 (Olympus, Japan) inverted fluorescence microscope at magnification 200 × [39]. For each treatment and control, 3 independent fluorescent images were captured and analyzed using ImageJ software [41].

### In vivo assessment

#### Animals and experimental design

The study involved 10 Czech Fleckvieh clinically healthy virgin heifers, aged 12 to 15 months. The heifers were housed in the experimental stable of the Veterinary Research Institute in Brno. The animals were housed in individual bonded stalls, separated by metal barriers, on a solid floor with straw bedding. They were fed ad libitum with commercial compound feed, hay, and silage, and had constant access to drinking water. All experiments and handling with animals were approved by the Branch Commission for Animal Welfare of the Ministry of Agriculture of the Czech Republic (permission number MZE-49165/2021-18134).

The heifers were divided into 2 groups of 5 animals each—experimental and control. After a 10-day adaptation period, intramammary infection with MRSA 2208 was applied in all quarters of the mammary gland in both groups. Heifers were sedated with xylazine and each teat end was carefully disinfected with gauze soaked in 70% ethanol. Two mL of bacterial suspension with infectious dose of 3.4 × 10^8^ CFU (colony forming unit)/quarter were injected to each quarter of all heifers (experimental and control groups). The application was performed through the teat orifice using a flexible polypropylene catheter with a rounded end (Catheter Dog 4FG, Covetrus, Portland, USA).

After 24 h of infection and the development of clinical symptoms of mastitis, a dose of 10 mL of SeTeNPs (149.70/263.95 mg/L in PBS) was administered to all quarters in the experimental group, while the control group received 10 mL of PBS, again using sterile catheter. Then, at 4 time intervals (1, 2, 3, and 7 d after the NPs/PBS application), samples of mammary gland lavage were collected, and the health status of the animals and mammary gland were checked. Lavages of mammary gland were performed by instillation of 5 mL of PBS and after a short massage of the udder, 3 mL of lavage was aspirated back into the syringe. Blood was collected (24 h and 168 h after the NPs/PBS application) from the coccygeal vein into tubes with heparin (whole blood sample) and a part was centrifuged at 1,300 × *g* for 15 min (plasma sample). Samples were stored at −80 °C until further use.

At the end of the experiment, all animals were treated with antimicrobials (intramammary treatment with Lineomam). After a withdrawal period, they were slaughtered at the abattoir, where samples of organs (liver, kidneys, lung, heart, spleen, and muscle) and mammary gland (including the teat) were collected. Before histological analysis, the slices of tissue samples were placed in 4% formaldehyde.

Despite being treated and confirmed free of *S. aureus*, the animals could not be reintegrated into the breeding programs after the infectious study. Consequently, they were slaughtered in accordance with standard procedures in slaughterhouses, as prescribed by current Czech legislation.

#### Evaluation of clinical status

The overall health of the heifers was checked by measuring body temperature, and the condition of the mammary gland was checked by adspection and palpation on the day of NPs/PBS application (d 0) and on the days of lavage collection (d 1, 2, 3, and 7). The clinical status of each mammary gland was evaluated by scoring: 1–normal (without symptoms of inflammation), 2–slight swelling, 3–moderate swelling (stiffness, soreness), 4–severe swelling (hardening, significant soreness) [42].

#### Microbiological assessment of lavage

The lavage samples were serially tenfold diluted in sterile PBS and 100 µL of the sample dilution was spread onto Columbia agar supplemented with 5% defibrinated sheep blood (LMS, Czech Republic) in duplicate. After incubation (24 h at 37 °C), colonies morphologically typical for *S. aureus* were counted and the number of CFU/mL was calculated**.**

#### Blood plasma metabolic profile

The levels of total proteins, albumin, cholesterol, alkaline phosphatase (ALP), alanine aminotransferase (ALT), aspartate aminotransferase (AST), creatine kinase (CK), creatinine, glucose, lactate, bilirubin, uric acid, triacylglycerol, and urea in blood plasma were measured at Department of Animal Morphology, Physiology and Genetics (Mendel University in Brno, Czech Republic). These measurements were conducted following their standardized protocols to ensure accuracy and consistency.

#### Flow cytometry analysis of differential cell count

Differential cell count was measured by flow cytometry (BriCyte E6, Mindray, Shenzhen, China) according to methods described by Sladek and Rysanek [43]. The cell suspensions were examined by flow cytometry with differentiation of 20,000 cells. The dot plots were evaluated using MR Flow software (Mindray, China).

#### Analysis of TNF-α

The concentration of TNF-α was measured by Bovine TNF-α ELISA kit (CUSABIO, Texas, USA) according to manufacturer’s instructions using ELISA reader Sunrise (Tecan, Austria). Data was assessed by software Kim32 v.5.15.

#### Histopathology analysis

The harvested tissue samples were fixed in 4% neutral buffered formalin for at least 48 h. The fixed samples were processed by standard histological methods using an automated tissue processor (Leica ASP6025, Leica Microsystems, Germany), after which they were embedded in paraffin blocks using a Leica EG 1150H paraffin embedding station (Leica Microsystems, Germany). Slices with a thickness of 3–5 µm were cut from each sample using a microtome (Leica RM2255, Leica Microsystems, Germany), stained with hematoxylin and eosin (H&E) and mounted on standard glass slides (Bammed, Czech Republic). The prepared samples were evaluated as light-microscopic images, which were obtained using an Olympus BX46 microscope (Olympus, Japan).

### Statistical evaluation

Data were analyzed by GraphPad Prism 8.0.1. (GraphPad Software, CA, USA). The *t*-test (specified in the text) or one-way ANOVA was used to detect significant differences between the control and SeTeNPs groups (statistical significance was declared at *P* < 0.05 and *P* < 0.01). The box plots were generated in RStudio via ggplot2 [44].

OpenAI’s ChatGPT [45] was used exclusively for language and stylistic editing.

## Results

### Characterization of SeTeNPs

**Fig. 1:**
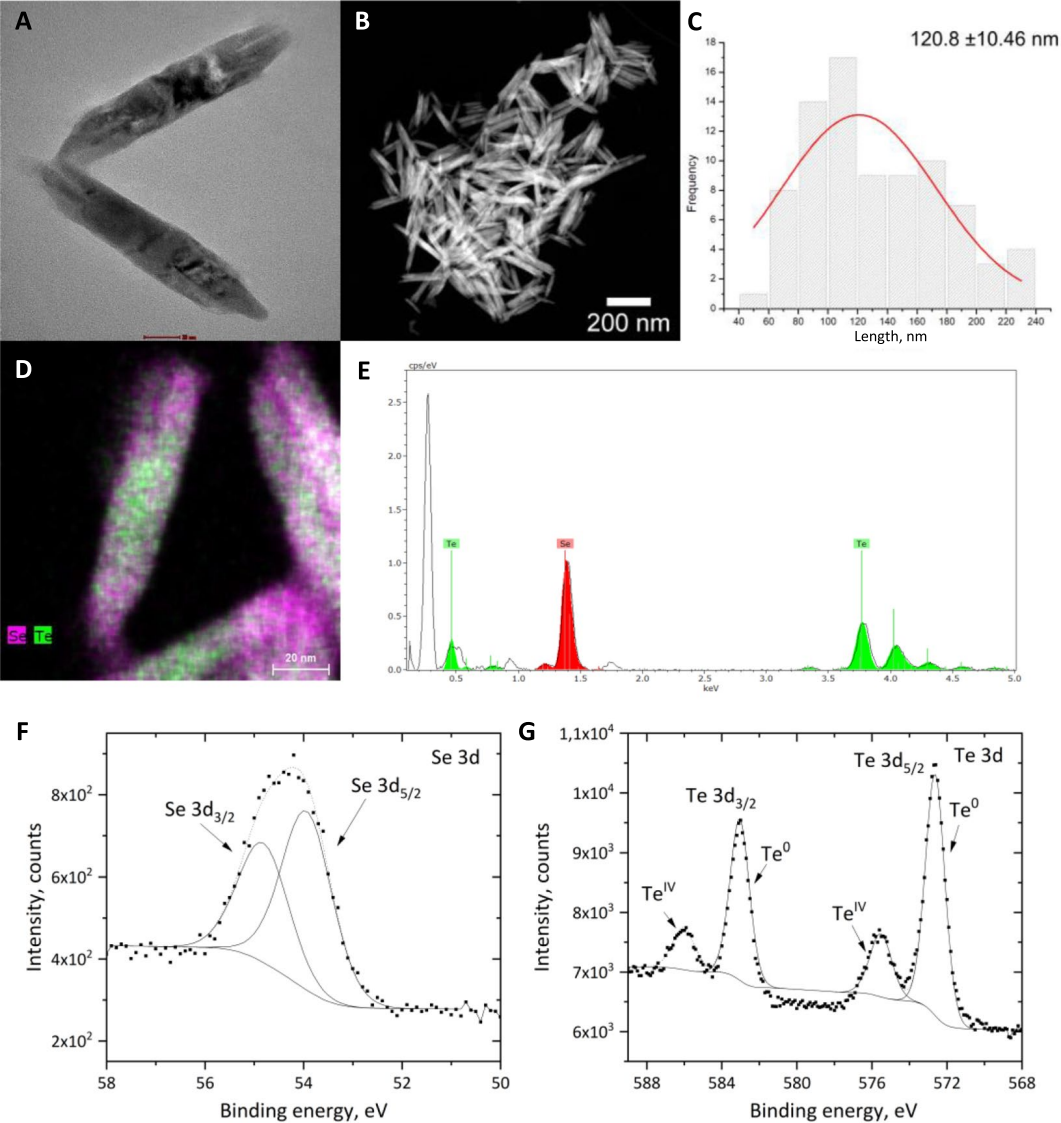
Structure and composition analysis of SeTeNPs. **A** Transmission electron microscopy (TEM) image of SeTeNPs. **B** Scanning transmission electron microscopy (STEM) image of SeTeNPs. **C** Histogram showing length distribution of SeTeNPs. **D** Elemental mapping of SeTeNPs. **E** Corresponding energy dispersive X-ray spectroscopy graph. **F** and **G** X-ray photoelectron spectroscopy (XPS) spectra in the Se 3d region (**F**) and Te 3d region of SeTeNPs (**G**)

An easy approach was devised to produce SeTeNPs. The procedure involves simultaneously heating equal concentrations of precursor salts, followed by the addition of reducing agents. The introduction of reducing agents leads to the formation of a blackish brown product, which is then washed through repeated cycles of centrifugation. The rod-like morphology with sharp edges, as observed through TEM (Fig. 1A), was further confirmed by scanning transmission electron microscopy (Fig. 1B), highlighting the uniformity of the NPs. The length of SeTeNPs was determined to be 120.80 ± 10.46 nm through image analysis performed on electron microscopy images (Fig. 1C). While quantitative analysis of the concentration of selenium and tellurium was measured using AAS (the concertation of Se was 299.4 mg/L and Te was 527.9 mg/L), qualitative analysis was performed by energy-dispersive X-ray spectroscopy (EDS). The elemental mapping of SeTeNPs (Fig. 1D and E) showed a uniform distribution of selenium and telluride in NPs. To gain insights into the chemical state and composition of SeTeNPs, XPS analysis was performed. Figure 1F and G present characteristic XPS spectra of Se 3d and Te 3d components, respectively. The Se 3d XPS spectrum (Fig. 1F) exhibited two distinct bands at 54.82 eV (Se 3d_3/2_) and 53.94 eV (Se 3d_5/2_). Similarly, the Te 3d deconvolution spectra (Fig. 1G) revealed 4 spectral bands at binding energies 572.63, 575.53, 583.04, and 586.04 eV, respectively [46]. The peaks at 572.63 eV and 583.04 eV were assigned to Te^0^, representing the elemental form of tellurium. On the other hand, the other 2 bands were attributed to Te^IV^, indicating the presence of tellurium oxide species in the prepared nanorods. The coexistence of these two states suggests that tellurium exists in both its elemental and oxide forms within the SeTeNPs [47]. The XRD pattern of SeTeNPs is shown in Fig. S1. The diffraction pattern corresponds to a hexagonal crystal structure with space group P3₁21 (152) (ICDD card No. 00–036–1452), indicating a well-defined crystalline phase. The predominant phase is based on tellurium’s hexagonal arrangement, with Se atoms partially substituting Te within the lattice. The average values of calculated lattice constants are a = b = 4.4521 Å, c = 5.352 Å. The Te and Se atoms are arranged in helical chains that spiral along the c-axis, which is characteristic of the hexagonal phases of this material. The stability of SeTeNPs morphology over a two-month period was confirmed by SEM (Fig. S2).

### Biological activity of SeTeNPs in vitro

#### Cytotoxic properties of SeTeNPs on eukaryotic cell line

**Fig. 2:**
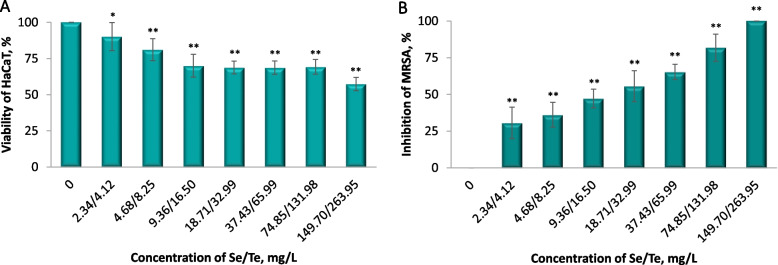
Toxicity of SeTeNPs to cells compared to untreated cells (control). **A** Viability of HaCaT cells treated with different concertations of SeTeNPs after 24 h. **B** Inhibition of MRSA after SeTeNPs treatment. Data represent the mean ± SD (*n* = 5), ^**^P < 0.01 and ^*^P < 0.05 indicate significant differences between the control (0 mg/mL) and the treated cells

The biocompatibility of synthesized SeTeNPs was studied on HaCaT cells. The results from MTT assay were plotted as the percentage of cell viability versus SeTeNPs concentration, results were presented as relative to the untreated cells (Fig. 2A). The SeTeNPs cytotoxicity was tested in the concentration range of 2.34–149.70 mg/L for Se and 4.12–263.95 mg/L for Te and presented concentrations refer to the concentrations of Se and Te. In comparison with the control group, the viability of the treated cells decreased with increasing concentration of NPs. This means that the lowest cell survival rate (57.4%, *P* < 0.01) was observed at the highest concentration of Se/Te (149.70/263.95 mg/L) and conversely the highest viability (90.2%, *P* < 0.05) showed cells treated with the lowest Se/Te concentration (2.34/4.12 mg/L).

#### Evaluation of antibacterial effect of SeTeNPs

**Fig. 3:**
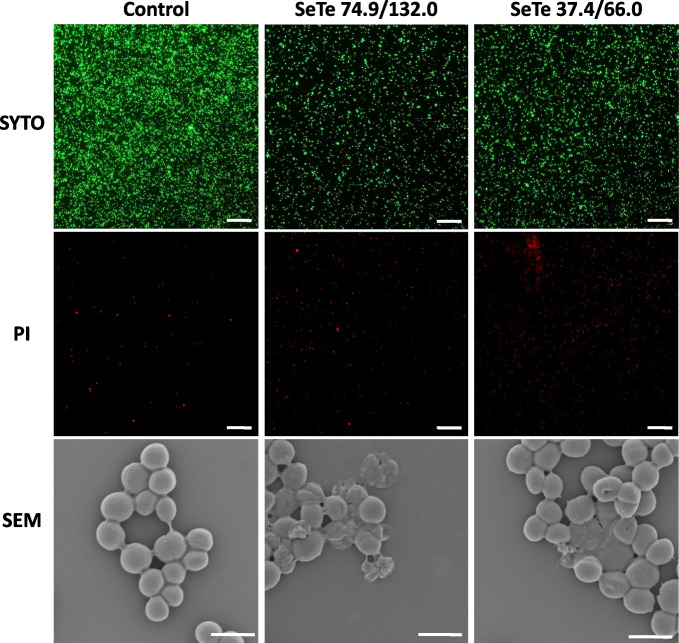
Evaluation of antibacterial activity using fluorescence microscopy and SEM on MRSA cells (control) and MRSA treated with two sub-inhibitory concentrations of SeTeNPs: Se 74.9 mg/L; Te 132.0 mg/L and Se 37.4 mg/L; Te 66.0 mg/L. The live and dead MRSA cells were visualized using green dye SYTO and dead cells using red dye PI. The scale bar for fluorescence microscopy is 100 µm. The scale bar for SEM micrographs is 1 µm. The brightness and contrast were processed equally across all micrographs

The measurements of the antibacterial activity were plotted as the percentage of MRSA inhibition versus SeTeNPs concentration, results were presented as relative to the untreated bacteria (control), and presented concentrations refer to the concentrations of Se and Te (Fig. 2B). MRSA growth was significantly inhibited after exposure to SeTeNPs and the reduction of growth was highly concentration dependent. The total growth inhibition (100%, *P* < 0.01) of MRSA was observed at the highest Se/Te concentration of 149.70/263.95 mg/L, this value was therefore considered as the MIC. Vice versa, the lowest growth reduction (30.6%, *P* < 0.01) was determined at the lowest Se/Te concentration (2.34/4.12 mg/L). Determination of antibacterial activity by fluorescence microscopy and SEM on MRSA cells exposed to 2 sub-inhibitory concentrations of SeTeNPs (74.9/132.0 mg/L and 37.4/66.0 of Se and Te) confirmed inhibitory effect compared to the untreated control (Fig. 3, quantification is shown in Fig. S3). The inhibitory effect of SeTeNPs on MRSA cells was visualized by fluorescence microscopy using the fluorescent probes SYTO 9 and PI, which selectively stain live and dead cells [48]. Bacteria were exposed to sub-inhibitory concentrations of SeTeNPs and untreated MRSA served as a control (Fig. 3). The control MRSA cells appeared predominantly green (demonstrating live cells) with few red spots (dead cells). When treated with SeTeNPs, the observations are in accordance with previous results. The higher sub-inhibitory concentration (74.9/132.0 mg/L) caused significant inhibition of cells (green spots, *P* < 0.01). Moreover, the presence of dead cells (red spots) increased compared to control (*P* < 0.01), which indicates the bacterial cell death due to the loss of membrane integrity due to NPs treatment. The concentration of 37.4/66.0 mg/L also reduced the bacterial growth (*P* < 0.05), however, the abundance of damaged cells was lower compared to higher SeTeNPs concentration.

The morphological changes of MRSA cells treated with SeTeNPs compared to control cells (untreated) were visualized by SEM (Fig. 3). The untreated MRSA cell surface was smooth and typical characters of the normal cells. On the contrary, the treated cells in both sub-inhibitory concentrations showed significant morphological alterations. At higher concentration (74.9/132.0 mg/L) were more abundant damaged and misshapen cells with lysis characteristics. Treatment with lower concentration (37.4/66.0 mg/L) exhibited less lysed cells, however there is observable crimped cell wall topography.

### Biological activity of SeTeNPs in vivo

#### Clinical signs

Acute mastitis was observed 24 h after infection in all 10 heifers during the clinical check. Mastitis was manifested by significant swelling, in some quarters even hardening of the udders, and soreness of the udders. The mammary secretion contained clots. A slight, but not significant (*P* = 0.071) decrease in the mammary score was observed in the SeTeNPs group on d 3 after NPs application compared to the control group (Table 1). Exposure to SeTeNPs had no significant effect on body temperature compared to the control group during the entire experiment (Fig. S4). The average body temperature of the cows throughout the experiment was 39.1 ± 0.2 °C for the control group and 39.2 ± 0.3 °C for the SeTeNPs group.

**Table 1:** Clinical status of mammary glands (mammary score) of the control group (*n* = 5) and the SeTeNPs group (*n* = 5) of cows at 4 time points: 1, 2, 3 and 7 d after the application of NPs/PBS

Days after NPs/PBS application	Control	SeTeNPs	*P* value
0	3.1 ± 1.0	3.3 ± 0.7	0.449
1	2.5 ± 0.9	3.0 ± 0.7	0.068
2	2.0 ± 1.0	1.8 ± 1.0	0.444
3	2.2 ± 1.1	1.6 ± 0.8	0.071
7	1.7 ± 1.0	1.2 ± 0.5	0.092

Data are expressed as mean ± standard deviation, differences between groups were estimated using Student’s *t*-test

#### Microbiological assessment of lavages of mammary glands

**Fig. 4:**
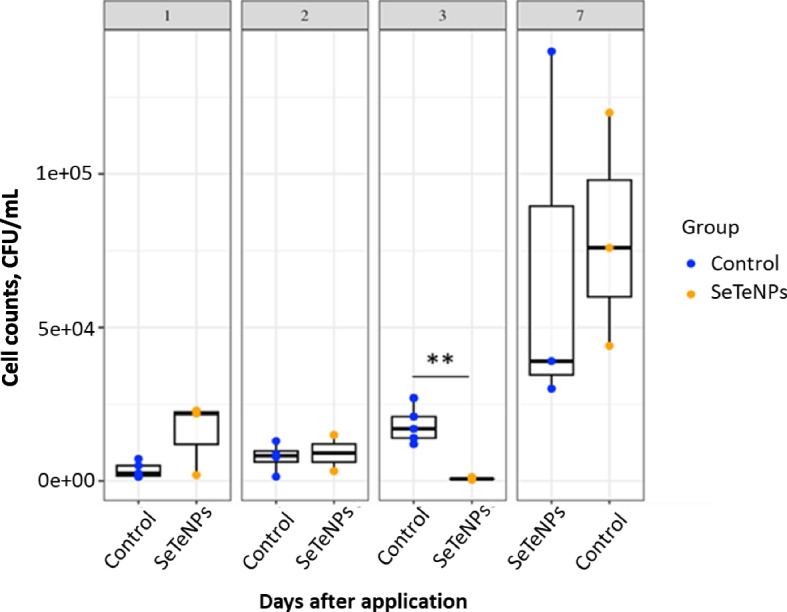
MRSA counts in the mammary gland lavages from the control group (*n* = 5) and the SeTeNPs group (*n* = 5) of cows throughout the experiment at 4 time points: 1, 2, 3 and 7 d after the application of NPs/PBS. ^**^P < 0.01 indicates significant differences between the control and the treated groups

Counts of MRSA were determined in the lavages of mammary glands at various time points after SeTeNPs/PBS treatment. Bacterial counts for time intervals 1, 2, 3, and 7 d after NPs/PBS application are shown in Fig. 4. Significant changes in MRSA colony counts (*P* < 0.01) were observed on day 3 after application of SeTeNPs compared to the untreated (PBS) control. No changes in the counts of MRSA colonies compared to the control were observed on the other days. Seven days after application, an increase in bacterial counts was observed.

#### The inflammatory status of the mammary gland

**Fig. 5:**
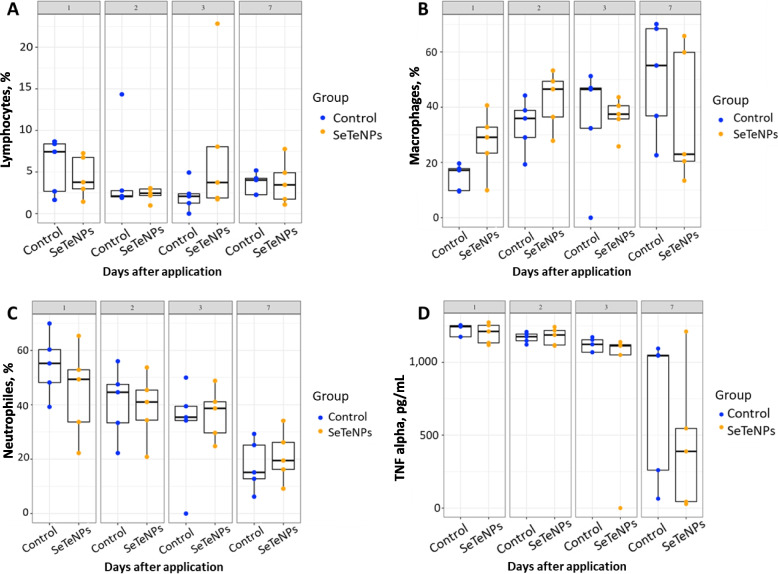
Differential cell count of (**A**) lymphocytes, (**B**) macrophages and (**C**) neutrophils; and (**D**) concentrations of TNF-alpha in lavages of the control group (*n* = 5) and the SeTeNPs group (*n* = 5) throughout the experiment at 4 time points: 1, 2, 3 and 7 d after application of NPs/PBS

The proportions of lymphocytes, macrophages and neutrophils are shown in Fig. 5. No significant effect of SeTeNPs treatment was observed. The proportions of the above cells indicate that the inflammatory response is proceeding in a normal manner. The increasing trend in the proportion of macrophages during the experiment is evident (Fig. 5B). A high number of neutrophils was observed at the beginning of the inflammation, which decreased by the end of the experiment (Fig. 5C). The TNF-alpha concentrations in the lavages have also not shown any significant changes when compared both groups (Fig. 5D). The highest TNF-alpha concentration was measured at the beginning of the experiment (Fig. 5D), which is consistent with the initial phase of inflammation.

#### Levels of different parameters in blood plasma

The concentrations of the tested parameters (protein, albumin, cholesterol, ALP, ALT, AST, CK, creatinine, glucose, lactate, bilirubin, uric acid, triacylglycerol, and urea) in blood plasma were analyzed 1 d and 7 d after NPs/PBS application (Table S2). No significant changes were observed on day 1. Seven days after the application of SeTeNPs/PBS, only the glucose concentration significantly increased (*P* < 0.05) compared to the control.

#### Histopathology examination

**Fig. 6:**
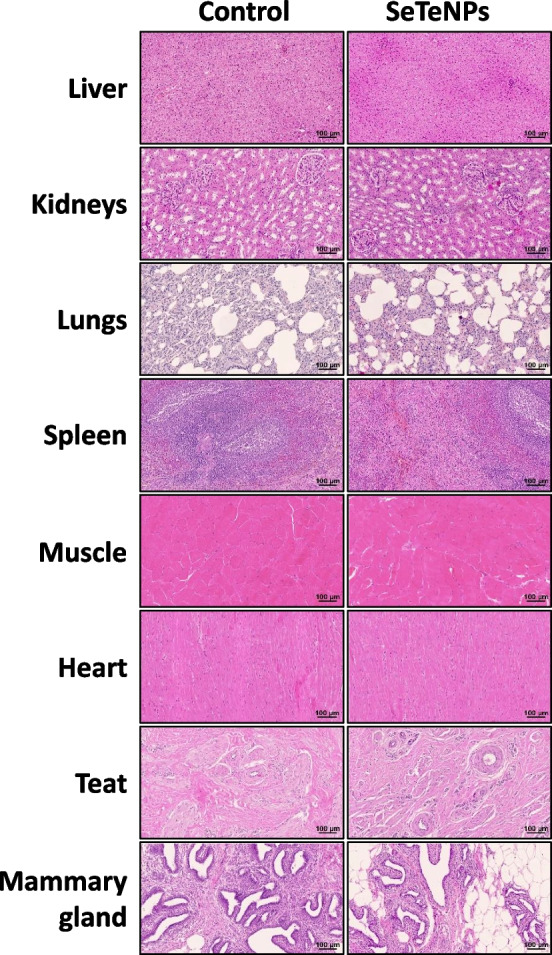
Histological examination of various organs from heifers (control group and the SeTeNPs group). Representative images of organs sections harvested one month post application were examined by H&E staining. Scale bar, 100 µm

For the assessment of long-term cytotoxicity of treatment with a single dose of SeTeNPs, the histopathology examination of various organs was performed one month post application. The samples were investigated by the veterinary pathologist, who was blinded to the duration and response of the treatment. All organs examined showed no significant pathological changes and no differences between the control and experimental group (Fig. 6).

## Discussion

### Physicochemical characterization of SeTeNPs

The literature directly addressing the synthesis of SeTe-based NPs remains limited, with only a few studies covering this area [46, 49]. Research to date has primarily focused on the individual synthesis of Se and Te NPs, with attention to factors like morphology, size, and stability. SeNPs are commonly synthesized under milder conditions, often using reducing agents such as ascorbic acid, resulting in spherical or rod-like shapes [50], whereas TeNPs typically require higher temperatures and stronger reducing agents to form nanorods or nanotubes [51]. Synthesizing SeTeNPs requires finding a balance of conditions suitable for both elements, presenting unique challenges due to their different optimal parameters. Our approach to creating stable SeTeNPs thus provides new insights into adjusting synthesis conditions, helping to fill the current gap in the literature and serving as a basis for future studies aiming to refine these methods.

The synthesis of SeTeNPs via the co-reduction of selenous and tellurous acids demonstrates an effective approach to producing nanomaterials with controlled size and morphology. The use of PVP as a stabilizing agent plays a crucial role in maintaining NPs uniformity and preventing agglomeration [52], which is essential to ensure consistent properties in nanomaterials. The polymeric structure of PVP provides steric hindrance, effectively capping the growing NPs and allowing for the control of their dimensions. Importantly, the SeTeNPs exhibited stability for several months, as confirmed by SEM analysis, further highlighting the effectiveness of this stabilization. The reaction conditions, particularly the temperature of 80 °C, are critical in promoting the reduction of both selenium and tellurium species. The elevated temperature likely enhances the kinetics of the reaction, facilitating the rapid conversion of precursor salts into NPs. Moreover, the rapid addition of sodium borohydride not only acts as a strong reducing agent but also contributes to the immediate formation of SeTeNPs, which is essential to achieve the desired rod-like morphology with sharp edges, as observed in subsequent characterizations.

### Biological activity of SeTeNPs in vitro

The application potential of antimicrobial NPs in agriculture is growing, therefore a toxicological evaluation prior to their utilization is essential [53]. To understand the biological effects of SeTeNPs on mammalian cells the cytotoxic properties of SeTeNPs were evaluated on the eukaryotic HaCaT cell line by MTT assay. It was found that the viability of HaCaT cells increased with decreasing SeTeNPs concentration and no half maximal inhibitory concentration (IC50) was observed within the tested concentration range. This finding correlates with previous evidence that selenium and tellurium NPs are considered cytocompatible (in a dose-dependent manner) to healthy cells, such as human dermal fibroblasts. While it mainly depends on the morphology and physico–chemical properties of the NPs [54, 55]. Low cytotoxicity on mammalian epithelial cells has been reported for Se and Te or in combination with other NPs [56–59]. The slight increase in cytotoxic activity of SeTeNPs could be associated with high reactivity of selenium and tellurium with intracellular antioxidant glutathione, which leads to elevated ROS levels [60, 61]. The cytotoxicity of SeNPs often escalates in a dose-dependent manner, as in the case of SeTeNPs, correlating with an increase in ROS production at higher doses [62]. Since the redox regulation and related signaling pathways of keratinocytes are different compared to other skin cells, e.g., fibroblasts, this could be the result of moderate sensitivity of HaCaT cells to SeTeNPs [63]. While in vitro studies on cultured cells are commonly used in experimental design, they often lack detailed insights into underlying mechanisms. As a result, translating in vitro nanotoxicity findings to more complex in vivo systems remains a challenge.

In the present study, the effect of SeTeNPs on MRSA cells was evaluated by the broth microdilution method and verified by fluorescence and electron microscopy, where SeTeNPs were found to be a potential antimicrobial candidate. A complete inhibition of MRSA was observed at the highest concentration of SeTeNPs (corresponds to MIC value), which gradually decreased with lower concentrations of NPs. Similar to other studies, both Se and Te NPs (bare or modified) reduced bacterial growth of various Gram-positive and Gram-negative bacteria [64–66], including MRSA [67, 68].

Fluorescence microscopy confirmed the inhibition of MRSA cells treated with SeTeNPs in both sub-inhibitory concentrations used. In both cases, an increased incidence of dead cells was observed as a result of the loss of membrane integrity due to NP treatment. The SEM examination also confirmed the morphological changes of the cells, showcasing in cell wall disruption as well as lysis of the MRSA cells themselves. Huang et al. found that the antibacterial effect of SeNPs against MRSA was size-dependent and this also results in the multimodal mechanism of action. The antibacterial properties were attributed to ROS generation, membrane potential disruption and the depletion of cellular ATP [69]. Oxidative stress mediated by elevated ROS production is considered in many studies to be the main cause of the antimicrobial effect of Se and Te NPs. ROS generation can lead to cell death through DNA damage, lipid peroxidation, and/or protein oxidation [70–72]. This oxidative stress on bacterial membrane causes leakage of cytoplasmic contents [28]. Zhang and coworkers reported morphological changes in *S. aureus* manifested by the folded and flattened membranes surrounded by cytoplasm with a hole on one side of the membrane, causing a large number of dead *S. aureus* cells, which were also detected by fluorescence microscopy. The authors suggested that the oxidative stress induced by ROS damaged the integrity of the cell membrane, leading to the collapse of the bacterial structures. In addition, DNA damage and disruption of transmembrane electron transport are probably one of the key mechanisms for the antibacterial effect of SeNPs [27]. The primary antibacterial action of TeNPs appears to involve membrane disruption. The rod-shaped TeNPs, with their sharp ends, interact with the membrane, leading to the leakage of intracellular enzymes and entry of extracellular chemicals. This membrane disruption is facilitated by electrostatic interactions between TeNPs and the cell surface, followed by the penetration of the sharp NP ends through the membrane [22]. The shape of NPs is a key factor in this process, as demonstrated with ZnONPs, where the rod-shaped ZnONPs show greater antibacterial activity than spherical or flower-like morphologies, likely due to enhanced penetration through bacterial cell membranes [73]. In our study, SeTeNPs were observed to alter membrane permeability and disrupt both cell walls and outer membranes, likely through a combination of ROS generation and direct physical interaction with the rod-shaped particles. This mechanism probably involves interactions with mammalian cells, as the complex relationship between NP properties and their biological effects across organisms can lead to unintended cytotoxicity. Therefore, it is essential to design NPs that selectively target bacterial cells while minimizing impact on mammalian cells [74]. Based on these results and previous studies, SeTeNPs appear to be a suitable substitute for antibiotics in the treatment of bovine mastitis.

### Biological activity of SeTeNPs in vivo

To verify the efficacy of SeTeNPs for potential therapeutic purposes in the mastitis treatment, a strain of MRSA was used in an in vivo experiment. *S. aureus* is one of the main causes of bovine mastitis worldwide [75] and this particular strain was selected because it originates from clinical mastitis, and is very difficult to eradicate with conventional antimicrobials due to its multidrug resistance. All 10 heifers managed to induce severe acute inflammation, manifested by stiffness and hardening of the udder. There was the evidence of MRSA attachment and multiplication in the mammary gland. SeTeNPs were administered intramammarily only after the development of clinical signs of mastitis, as intended for their use in herds. On the first and second day after their application, no differences in MRSA counts or the severity of inflammation were observed compared to the control group. However, on the third day, a significant reduction in MRSA counts was observed in the experimental group, accompanied by a noticeable improvement in the condition of the mammary gland. The inhibition effect demonstrated within the in vitro experiments was thus manifested, leading to a faster resolution of inflammation. Numerous studies have investigated the inhibitory effects of various NPs on mastitis-causing bacteria in vitro. These include metal NPs [76], or polymer NPs [77], and/or NPs in combination with antibiotics [78]. However, only a limited subset of these studies has undergone in vivo testing, at least in rodent models [79–81]. To the best of the authors' knowledge, selenium or tellurium NPs have not been tested for the treatment of mastitis infection in bovine model.

Although the condition of the mammary gland gradually improved and the inflammation subsided with each subsequent day, a renewed increase in MRSA counts was observed on the seventh day in some heifers from both groups. The elevated counts of MRSA contradicted the clinical signs, as the mammary score reached its lowest level in both groups seven days after treatment application. At this point, however, it would be necessary to intervene with a second dose of NPs to suppress the growth of *S. aureus* again, including disrupting any biofilm that may have formed, thereby making the bacteria accessible to the immune system cells. *S. aureus* often forms a biofilm, a structure that protects the bacteria against the effects of antimicrobials and the immune system, thus making the treatment more complicated [82]. The presence of biofilms can reduce the effectiveness of conventional antibiotics, and prolonged or increased doses of antibiotics raise the risk of developing antibiotic resistance [83]. An alternative approach could involve using Se or Te-based NPs, which have shown the ability to disrupt biofilms of various bacterial species, including those associated with bovine mastitis [66, 84, 85]. However, further investigation is needed to understand how biofilm-producing strains will respond to additional doses of SeTeNPs. Administering repeated doses of NPs for infection treatment has yielded promising results, including in enhancing therapeutic effects against MRSA infections [86]. After administering a repeated dose of NPs, it becomes crucial to monitor the impact on surrounding tissues as well as the biodistribution and bioaccumulation of these novel materials [87].

Evaluation of the effect of NPs application on the heifer health status is a crucial aspect of assessing their potential health risk. In experimental cows, the inflammatory response in the mammary gland (at the site of NP administration) was comparable to that of the control group, indicating no exacerbation of infection-induced inflammation. In fact, the literature suggests that SeNPs can inhibit inflammatory factors and may possess anti-inflammatory properties [88, 89]. When SeTeNPs are introduced into the biological system, the Se atoms from NPs may be utilized by cells for the synthesis of selenoproteins. Immune response studies indicate that treatment of mammalian cells with SeNPs leads to the upregulation of antioxidant selenoproteins such as glutathione peroxidase and thioredoxin reductase [90]. This increased expression enhances cellular antioxidant capacity, potentially reducing oxidative stress and inflammation. Further studies are needed to better understand the bioavailability of Se from NPs and the extent to which they contribute to the upregulation of selenoproteins compared to other Se sources [91]. Histopathological examination of the major organs showed no tissue alterations after the treatment. The mammary gland tissue also exhibited no histological changes. These findings indicate that SeTeNPs do not induce pathological effects, even at the application site. This observation is further corroborated by the unaffected metabolic profile of the blood plasma collected from heifers throughout the experimental phase. Se-based NPs demonstrate good biocompatibility in vivo, highlighting their potential as promising antibacterial, antitumour, and/or anti-inflammatory agents [92–94]. However, the employment of SeNPs as a therapeutic agent depends on the optimum dose and application. High doses of SeNPs or, vice versa, long-term administration of low doses could lead to Se toxicity in mammals [95, 96]. Similar outcomes show also Te-based NPs, where antibacterial TeNPs appear to be biocompatible in model organisms and can thus be considered for various biomedical applications [97–99]. Additionally, a study by Chen and colleagues demonstrated the significant reduction in toxicity and lethality in mice after the TeNPs were doped with Se. These synthesized TeSe nanomaterials exhibited superior biocompatibility and stability compared to TeNPs alone [100].

Considering that SeTeNPs have demonstrated the ability to inhibit MRSA isolated from mastitis, they likely offer therapeutic potential against other *S. aureus*-related infections, particularly those involving resistant strains. For instance, Se-based NPs have shown promise in applications such as wound healing, respiratory infection treatments, or a coating of orthopedic medical devices preventing infection at surgical site [68, 101, 102]. This suggests that SeTeNPs could serve as a versatile tool against resistant bacterial infections across human and veterinary medicine, broadening therapeutic options in areas where conventional treatments are limited. The application of NPs in livestock is emerging as a potential breakthrough, aiming to address challenges in infection control, growth promotion, and immune enhancement. Besides antimicrobial properties against pathogens, NPs (such as Zn or Se) are also used as dietary supplements to boost immune response and improve growth rates in livestock such as poultry, cattle, and swine [103, 104]. Currently, only a limited number of nanoparticle applications are approved for livestock, mainly as dietary supplements or as antimicrobial additives in animal feed. Most antimicrobial NPs remain in experimental stages due to challenges in establishing safe usage profiles. Regulation agencies, such as the European Food Safety Authority (EFSA), emphasize the necessity of ensuring that no NPs residues remain in animal products, like milk, meat, or eggs, to protect consumers [105]. For instance, in the case of using SeTeNPs to treat mastitis in dairy cows, it would be essential to confirm that NPs residues are not present in the milk. Comprehensive studies are necessary to confirm that NPs pose no long-term health risks to both animals and humans [106]. This includes investigating whether NPs accumulate in animal tissues or impact their microbiome over time. Another point, which is very important to attend to, is their fate once they get into the environment and their impact on soil and water ecosystems. In addition to the aforementioned, introducing nanomaterials into agriculture requires careful consideration of several factors: achieving reproducibility in their production (including consistent quality and particle size), scaling production from lab to commercial volumes, and ensuring cost-effectiveness and competitiveness with existing commercial products [107].

## Conclusions

The novel SeTeNPs have been evaluated as a treatment for acute bovine mastitis caused by a strain of MRSA. The study results suggest that administering SeTeNPs had a positive therapeutic effect on mastitis. For potential therapeutic use, multiple doses are required at specific intervals, and the dosing regimen needs to undergo further testing. Regarding safety, SeTeNPs have demonstrated biocompatibility and did not cause any adverse changes at the site of administration. Since this study was conducted on a limited number of samples, it should be considered a proof of concept for the action of our synthesized NPs. Nevertheless, it can be concluded that the SeTeNPs are antibacterial and biocompatible even in the bovine model, indicating their potential as a novel therapeutic agent against mastitis, pending further testing. Given the potential of SeTeNPs to reduce MRSA infections in animals through mechanisms that prevent bacteria from easily developing resistance, these NPs present a promising approach in combating bacterial resistance. Future plans include evaluating SeTeNPs against a broader spectrum of resistant pathogens beyond MRSA, potentially extending to biofilm-associated infections prevalent in agriculture and healthcare. Additionally, combining SeTeNPs with conventional antibiotics could enhance the effectiveness of existing treatments by creating a synergistic effect, thereby reducing the selective pressure on bacteria and delay the resistance development.

## Supplementary Information

Additional file 1: Table S1 Antimicrobial susceptibility of strain MRSA 2208. Table S2 Plasma levels of different parameters in the control group (*n* = 5) and the SeTeNPs group (*n* = 5) of cows measured 1 d and 7 d after NPs/PBS application. Fig. S1 The XRD diffractogram of SeTeNPs. Fig. S2 SEM images of SeTeNPs during a stability study: November (left) and January (right). Fig. S3 Live and dead MRSA cells were visualized by using SYTO and PI. The counts of (A) live (green) and (B) dead (red) cells were obtained using ImageJ analysis. MRSA was treated with two sub-inhibitory concentrations of SeTeNPs: Se 74.9 mg/L; Te 132.0 mg/L and Se 37.4 mg/L; Te 66.0 mg/L, Data represent the mean ± SD (*n* = 3). ^*^P < 0.05, ^**^P < 0.01 (compared to controls, 0 mg/mL). Fig. S4 Body temperature measurements of the control group (*n* = 5) and the SeTeNPs group (*n* = 5) throughout the duration of the experiment (1, 2, 3 and 7 d after the application of NPs/PBS).

## Data Availability

All data generated or analysed during this study are included in this published article. Data are available on the department share drive and are available from the corresponding author on the reasonable request.

## Abbreviations

ALP: Alkaline phosphatase
ALT: Alanine transaminase
AST: Aspartate transaminase
CK: Creatine kinase
HaCaT: Human epidermal keratinocyte line
HRTEM: High-resolution transmission electron microscopy
MIC: Minimum inhibitory concentration
MRSA: Methicillin-resistant *S. aureus*
MTT: 3-(4,5-Dimethylthiazol-2-yl)-2,5-diphenyltetrazolium bromide
NPs: Nanoparticles
PBS: Phosphate-buffered saline
PI: Propidium iodide
PVP: Polyvinylpyrrolidone
SEM: Scanning electron microscopy
TEM: Transmission electron microscopy
TNF: Tumor necrosis factor
TSB: Tryptone soy broth
XPS: X-ray photoelectron spectroscopy
XRD: X-ray diffraction
