# Supplementary material for: In vivo evaluation of selenium-tellurium based nanoparticles as a novel treatment for bovine mastitis
Source: J Anim Sci Biotechnol. 2024 Dec 20;15:173. doi: 10.1186/s40104-024-01128-y (PMC11660941; doi:10.1186/s40104-024-01128-y)
Supplement: Supplementary file 1 — Additional file 1: Table S1 Antimicrobial susceptibility of strain MRSA 2208. Table S2 Plasma levels of different parameters in the control group (n = 5) and the SeTeNPs group (n = 5) of cows measured 1 d and 7 d after NPs/PBS application. Fig. S1 The XRD diffractogram of SeTeNPs. Fig. S2 SEM images of SeTeNPs during a stability study: November (left) and January (right). Fig. S3 Live and dead MRSA cells were visualized by using SYTO and PI. The counts of (A) live (green) and (B) dead (red) cells were obtained using ImageJ analysis. MRSA was treated with two sub-inhibitory concentrations of SeTeNPs: Se 74.9 mg/L; Te 132.0 mg/L and Se 37.4 mg/L; Te 66.0 mg/L, Data represent the mean ± SD (n = 3). *P < 0.05, **P < 0.01 (compared to controls, 0 mg/mL). Fig. S4 Body temperature measurements of the control group (n = 5) and the SeTeNPs group (n = 5) throughout the duration of the experiment (1, 2, 3 and 7 d after the application of NPs/PBS). [file 40104_2024_1128_MOESM1_ESM.docx]

**Supplementary information**

**Characterization of the MRSA infectious strain**

Strain MRSA (no. VUVeL 2208) was isolated from mastitis in May 2017 in the Czech Republic. The detailed description of the strain's sensitivity to antimicrobials is shown in **Table S1**.

*Table S1 Antimicrobial susceptibility of strain MRSA 2208*

|  | **PNC** | **AMC** | **KF** | **EFT** | **CLI** | **GEN** | **NEO** | **STR** | **SXT** | **ENR** | **TET** | **RIF** | **FOX** | **OXA** |
| --- | --- | --- | --- | --- | --- | --- | --- | --- | --- | --- | --- | --- | --- | --- |
| **Broth dilution method** | R | R | S | R | S | R | S | R | S | S | R | R | R | R |
| **MIC (mg/l)** | >8 | 16 | 4 | 32 | ≤0.125 | ≤128 | ≤4 | 1024 | 2 | 0.13 | >32 | >4 | >16 | >2 |

*R = resistant; I = intermediate; S = sensitive. Tested antimicrobials: PNC = penicillin, AMC = amoxicillin/clavulanic acid 2/1, AMP = ampicillin, KF = cephalothin, EFT = ceftiofur, CLI = clindamycin, GEN = gentamicin, NEO = neomycin, STR = streptomycin, SXT = trimethoprim/sulfamethoxazole 1/19, ENR = enrofloxacin, TET = tetracycline, RIF = rifaximin, FOX = cefoxitin, OXA = oxacillin, ERY = erythromycin*

*Table S2 Plasma levels of different parameters in the control group (n = 5) and the SeTeNPs group (n = 5) of cows measured 1 day and 7 days after NPs/PBS application*

| **Days after NPs application** | **Group** | **Protein (g/l)** | **Albumin (g/l)** | **Cholesterol (mmol/l)** | **ALP (µkat/l)** | **ALT (µkat/l)** | **AST (µkat/l)** | **CK (µkat/l)** | **Creatinine (µmol/l)** | **Glucose (mmol/l)** | **Lactate (mmol/l)** | **Bilirubin (µmol/l)** | **Uric acid (µmol/l)** | **Triacylglyce-rol (mmol/l)** | **Urea (mmol/l)** |
| --- | --- | --- | --- | --- | --- | --- | --- | --- | --- | --- | --- | --- | --- | --- | --- |
| **1** | **Control** | 69.41±2.51 | 37.8±1.67 | 1.51±0.5 | 1.67±0.24 | 0.34±0.08 | 1±0.14 | 8.87±9.63 | 106.4±16.99 | 5.79±1.84 | 0.6±0.15 | 2.19±1.9 | 12.84±2.84 | 0.1±0.03 | 4.52±1.66 |
|  | **SeTeNPs** | 73.84±3.79 | 37.22±1.72 | 1.7±0.11 | 1.84±0.47 | 0.33±0.04 | 0.89±0.18 | 7.15±6.38 | 109.62±7.49 | 7.39±2.11 | 0.8±0.18 | 1.57±1.2 | 14.34±1.96 | 0.09±0.02 | 4.06±0.65 |
| **7** | **Control** | 74.98±7.54 | 38.03±1.17 | 1.51±0.41 | 1.71±0.39 | 0.34±0.09 | 0.99±0.14 | 5.46±4.69 | 103.85±11.17 | 5.77±1.56 | 0.71±0.34 | 0.66±0.22 | 15.98±3.08 | 0.08±0.03 | 3.16±0.76 |
|  | **SeTeNPs** | 72.92±5.02 | 36.7±0.98 | 1.62±0.1 | 2.09±0.17 | 0.33±0.01 | 0.98±0.27 | 4.68±1.79 | 104.47±11.56 | 9.81±1.08* | 0.64±0.19 | 0.87±0.59 | 12.31±2.35 | 0.1±0.04 | 3.3±1.1 |

*Data are expressed as mean ± standard deviation, (*) means significance versus control group (P = 0.045). ALP = alkaline phosphatase, ALT = alanine aminotransferase, AST = aspartate aminotransferase), CK = creatine kinase*


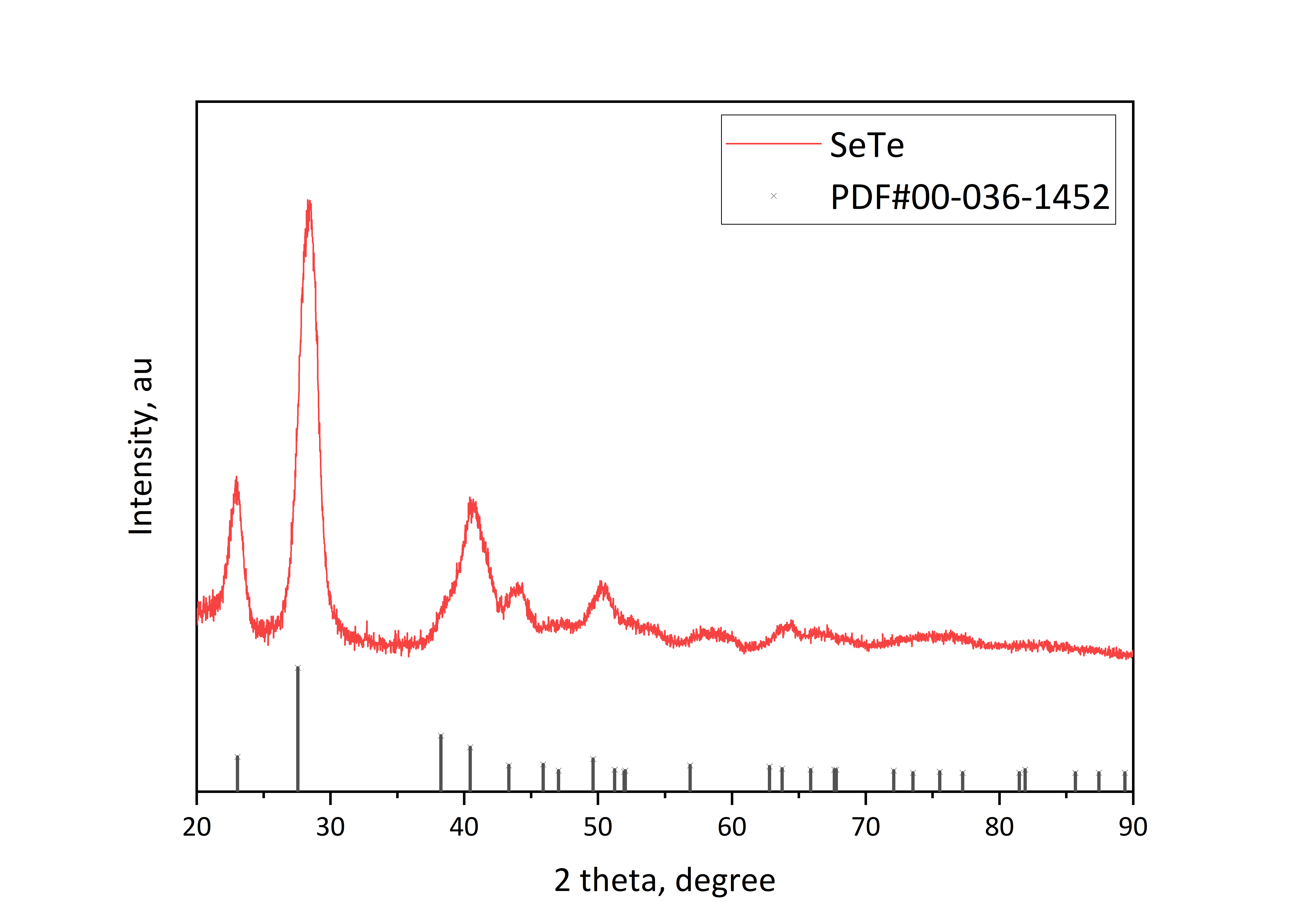


*Fig. S1 The XRD diffractogram of SeTeNPs*


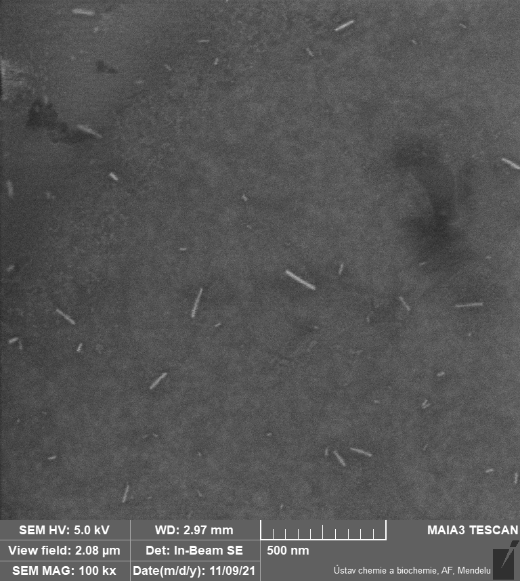

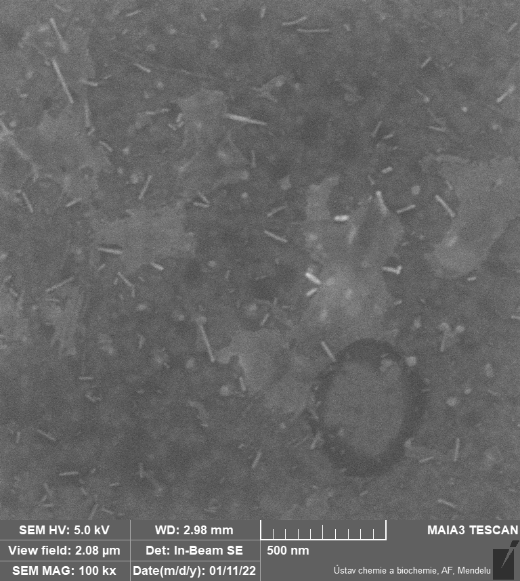


*Fig. S2 SEM images of SeTeNPs during a stability study: November (left) and January (right)*

*
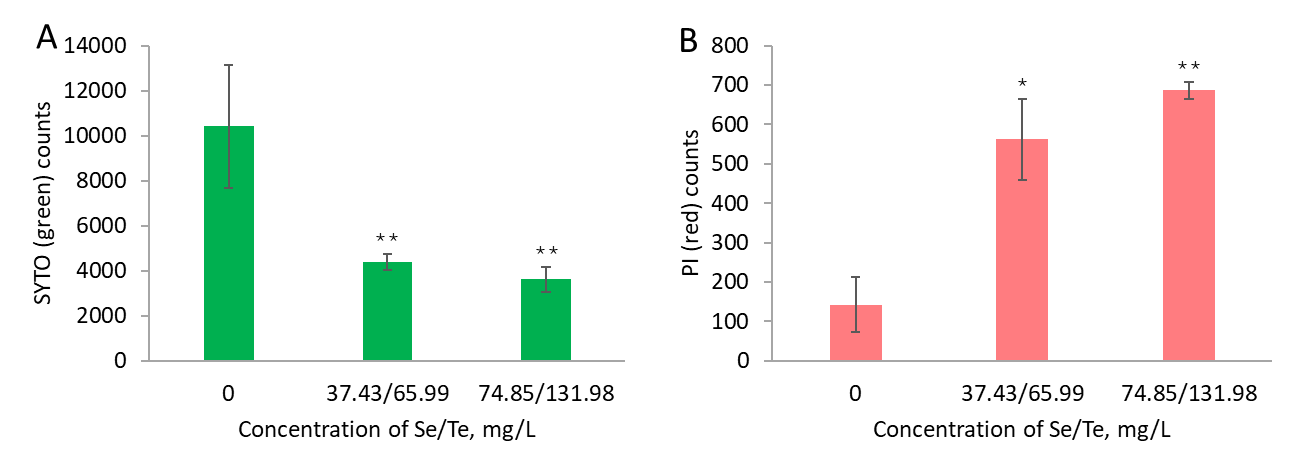
*

*Fig. S3 Live and dead MRSA cells were visualized by using SYTO and PI. The counts of (A) live (green) and (B) dead (red) cells were obtained using ImageJ analysis. MRSA was treated with two sub-inhibitory concentrations of SeTe NPs: Se 74.9 mg/L; Te 132.0 mg/L and Se 37.4 mg/L; Te 66.0 mg/L, Data represent the mean ± SD (n = 3). *P < 0.05, **P < 0.01 (compared to controls, 0 mg/mL)*


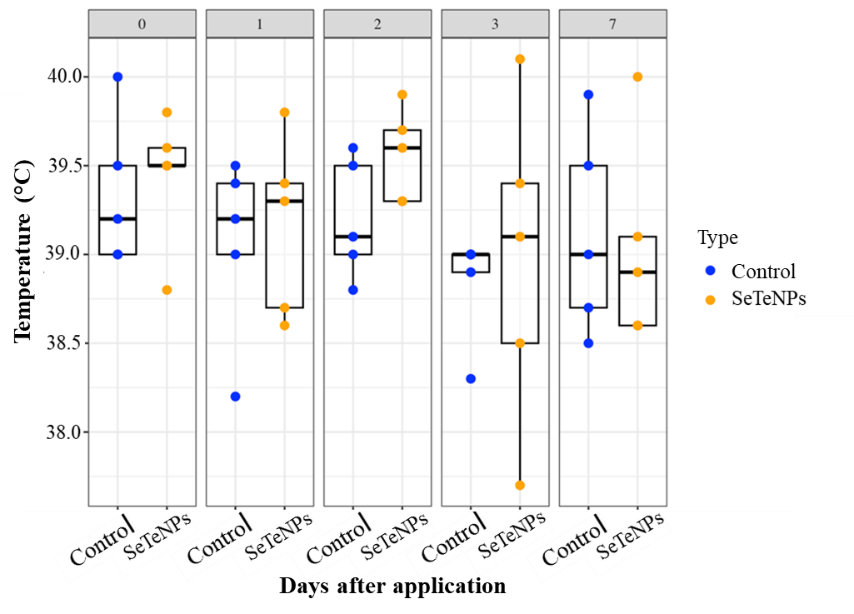


*Fig. S4 Body temperature measurements of the control group (n = 5) and the SeTeNPs group (n = 5) throughout the duration of the experiment (1, 2, 3 and 7 days after the application of NPs/PBS)*
